# NRX-101 (D-cycloserine plus lurasidone) vs. lurasidone for the maintenance of initial stabilization after ketamine in patients with severe bipolar depression with acute suicidal ideation and behavior: a randomized prospective phase 2 trial

**DOI:** 10.1186/s40345-023-00308-5

**Published:** 2023-08-13

**Authors:** Andrew Nierenberg, Philip Lavin, Daniel C. Javitt, Richard Shelton, Michael T. Sapko, Sanjay Mathew, Robert E. Besthof, Jonathan C. Javitt

**Affiliations:** 1 Massachusetts General Hospital, Boston, MA USA; 2 Lavin Consulting LLC, Framingham, MA USA; 3 Columbia University Vagelos College of Physicians and Surgeons, New York, NY USA; 4 NRx Pharmaceuticals, Inc, 1201 N Market St Suite 111, Wilmington, DE 19801 USA; 5 University of Alabama at Birmingham, Birmingham, AL USA; 6 Baylor College of Medicine, Houston, TX USA; 7 Johns Hopkins University, Baltimore, MD USA

**Keywords:** Bipolar disorder, Depression, Suicidal ideation, Ketamine, Lurasidone Hydrochloride, Cycloserine

## Abstract

**Background:**

We tested the hypothesis that, after initial improvement with intravenous ketamine in patients with bipolar disorder (BD) with severe depression and acute suicidal thinking or behavior, a fixed-dose combination of oral D-cycloserine (DCS) and lurasidone (NRX-101) can maintain improvement more effectively than lurasidone alone.

**Methods:**

This was a multi-center, double-blind, twostage, parallel randomized trial. Adult BD patients with depression and suicidal ideation or behavior were infused with ketamine or saline (Stage 1); those who improved were randomized to a fixed-dose combination of DCS and lurasidone vs. lurasidone alone (Stage 2) to maintain the improvement achieved in Stage 1. Depression was measured by the Montgomery Åsberg Depression Rating Scale (MADRS), and suicidal thinking and behavior was measured by the Columbia Suicide Severity Rating Scale (C-SSRS); global improvement was measured by the clinical global severity scale (CGI-S). Clinicaltrials.gov NCT02974010; Registered: November 22, 2016.

**Results:**

Thirty-seven patients were screened and 22 were enrolled, randomized, and treated. All 22 patients treated in Stage 1 (17 with ketamine and 5 with saline) were enrolled into Stage 2, and 11 completed the study. The fixed-dose combination of DCS and lurasidone was significantly more effective than lurasidone alone in maintaining improvement in depression (MADRS LMS Δ-7.7; p = 0.03) and reducing suicidal ideation, as measured by C-SSRS (Δ-1.5; p = 0.02) and by CGI-SS (Δ-2.9; p = 0.03), and with a non-statistically significant decrease in depressive relapse (0% vs. 40%; p = 0.07). This sequential treatment regimen did not cause any significant safety events and demonstrated improvements in patient-reported side effects.

**Conclusions:**

Sequential treatment of a single infusion of ketamine followed by NRX-101 maintenance is a promising therapeutic approach for reducing depression and suicidal ideation in patients with bipolar depression who require hospitalization due to acute suicidal ideation and behavior. On the basis of these findings, Breakthrough Therapy Designation was awarded, and a Special Protocol Agreement was granted by the FDA for a registrational trial.

**Supplementary Information:**

The online version contains supplementary material available at 10.1186/s40345-023-00308-5.

## Background

Bipolar disorder (BD) has a lifetime prevalence of 4.4% in adults in the United States, and the risk of acute suicidal ideation and behavior (ASIB) is uniquely high in patients during bipolar depressive episodes (Merikangas et al. 2007; Nierenberg, Gray, and Grandin 2001). It is estimated that up to 50% of individuals with BD may have suicidal behavior over their lifetime (Pallaskorpi et al. 2017), and between 11% and 19.9% (Holma et al. 2014) will die from suicide. Patients with BD are 20–30 times more likely to have suicidal behavior than the general population (Pompili et al. 2013), and the overall rate of death by suicide among patients with BD is approximately 10-fold greater than that of the general population (Schaffer et al. 2015).

Despite its lethal characteristics, there is no approved pharmacologic treatment for patients with bipolar depression in the presence of ASIB. Electroconvulsive therapy (ECT), often combined with inpatient psychiatric care, remains the only FDA-approved treatment for patients with ASIB in bipolar depression. Several combined dopamine D_2_ receptor and serotonin 5-HT_2A_ receptor antagonists—such as olanzapine/fluoxetine, quetiapine, lurasidone, cariprazine, and lumateparone—have demonstrated efficacy in treating bipolar depression; however, none have been shown to decrease ASIB, and all have an FDA-mandated warning regarding a potential to increase the risk of suicide (Kadakia et al. 2021; Loebel et al. 2014).

The discovery that ketamine has a rapid and profound effect on depression and suicidality (Berman et al. 2000) has led to broad recognition that glutamate system and N-methyl D-aspartate Receptor (NMDAR) antagonists may also play an important role in the treatment of depression and suicidality. Ketamine has since been shown in multiple randomized clinical trials to induce nearly immediate remission from depressive symptoms and from suicidal ideation (Wilkinson et al. 2018; Erhardt et al. 2013). However, the clinical effect of a single ketamine infusion is demonstrated to diminish very quickly, e.g., three days post-intravenous dosing (Zarate et al. 2012; Abbar et al. 2022) and two days post-intranasal dosing (Canuso et al. 2018). Moreover, ketamine is an addictive controlled substance that causes hallucinations, has known neurotoxicity, and exhibits abuse potential.

D-cycloserine (DCS) is a broad-spectrum antibiotic approved for the treatment of tuberculosis that has been used in millions of individuals without report of significant safety concerns or abuse potential. DCS was serendipitously found to have antidepressant effects by Crane (Crane 1959), which were subsequently confirmed in a placebo-controlled trial (Crane 1961). However, further development as an antidepressant was limited by psychotomimetic side effects.

In humans, DCS primarily manifests NMDA agonist effects at doses below 50 mg/d, whereas it primarily manifests NMDA antagonist effects at doses of > 500 mg/d. DCS at a target dose of > 500 mg/d has been shown to have a clinical antidepressant effect when administered to patients with treatment-resistant depression as an adjunct to SSRIs (Heresco-Levy et al. 2013). DCS sustained the antidepressant effect of ketamine in an open label trial in patients with treatment-resistant bipolar depression (Kantrowitz et al. 2015). DCS has further been demonstrated to maintain remission from suicidality after ketamine infusion, an effect that is more pronounced among patients with bipolar depression (Chen et al. 2019).

DCS is a potentially attractive oral antidepressant candidate because, unlike direct NMDA channel-blocking agents, it has shown no potential for neurotoxicity or abuse. Moreover, in nonclinical models, it has been shown to decrease akathisia induced by D_2_/5-HT_2A_-targeted drugs, including atypical antipsychotic agents such as lurasidone (Javitt 2016).

In this study, we tested the hypothesis that a fixed-dose combination of oral DCS and lurasidone (NRX-101) could better maintain initial stabilization from severe depression and ASIB in acutely depressed BD patients than could lurasidone alone following improvement with a single infusion of ketamine.

## Methods

### Study design

The study was conducted in accordance with the ethical principles as set out in the Declaration of Helsinki and its amendments and in compliance with the approved protocol of the United States Food and Drug Administration (FDA). The protocol (NCT02974010) and its associated Informed Consent Agreement were reviewed and approved by the central Institutional Review Board (IRB) of this study, Schulman IRB (now Advarra, Inc). Participants were evaluated to ensure that they were capable of understanding the nature of this study and its potential risks, discomforts, and benefits. All participants provided written informed consent.

This was a multi-center, double-blind, twostage, parallel randomized trial to evaluate the efficacy of ketamine, compared to saline, in the rapid improvement of patients with severe BD and ASIB (Stage 1) and the efficacy of NRX-101, compared to lurasidone, in maintaining the improvement achieved in Stage 1 (Stage 2). There were separate randomizations for Stage 1 and Stage 2.

This study was originally designed for a larger sample size but was terminated prior to full enrollment to accommodate a Special Protocol Agreement (SPA) with FDA wherein the primary endpoint scale was changed from the Bipolar Inventory of Symptoms Scale (BISS)-derived Montgomery-Åsberg Depression Rating Scale (MADRS) (BDM) to the traditional MADRS 10-item scale. This request was made by FDA to facilitate post-hoc comparison of NRX-101 results with placebo results already on file at FDA. The FDA additionally requested a separation of Stage 1 and Stage 2 for the SPA protocol. This study enrolled, randomized, and treated 22 patients prior to its early termination.

### Recruitment and eligibility

The study was conducted at one academic site (UAB, Birmingham) and two community hospitals. Key eligibility criteria included adults 18 to 65 years of age diagnosed with BD (Bipolar Disorder I or II) by a site psychiatrist according to DSM-5 criteria, supported by the MINI 7.0.2, and confirmed by remote, independent raters; a score equal to or greater than 20 on the MADRS items of the BISS; suicidal ideation or behavior of sufficient severity to meet the requirements for a score of 4, or 5 on the Columbia Suicide Severity Rating Scale (C-SSRS); able to understand and provide informed consent; deemed likely to comply with study protocol and communicate adverse events (AEs) and other clinically important information; and agreed to be hospitalized to complete screening and initiate experimental treatment. Concurrent psychotherapy and hypnotic therapy were allowed if the therapy had been stable for at least three months (for psychotherapy) or four weeks (for hypnotic therapy) prior to screening and if the therapy was expected to remain stable during the study.

Key exclusion criteria included females who were pregnant, breastfeeding, or of childbearing potential and were unwilling to use one of the specified forms of birth control during the study and individuals who had any of the following: moderate or severe substance use disorder within the 12 months prior to screening, a lifetime history of phencyclidine or ketamine drug use, prior failed use of ketamine for depression, any history of psychotic symptoms when not in an acute bipolar mood episode, any major psychiatric disorder that was clinically predominant to BD at screening or with BD as the primary or secondary focus of treatment at any time within six months prior to screening, dementia, delirium, amnestic, or any other cognitive disorder or clinically significant neurological disorder. Additional exclusion criteria are shown on clinicaltrials.gov.

### Study intervention

The Investigator, Patient, and Study Staff were blinded to treatment assignment, except for the unblinded pharmacist who prepared the Stage 1. Packaging and labeling of the study drugs were performed to ensure blinding throughout the study by providing uniquely assigned kit numbers per randomized patient and identical appearance of NRX-101 and lurasidone comparator capsules.

For Stage 1, the experimental drug was ketamine hydrochloride (PAR Sterile Products; Rochester, MI) 0.5 mg/kg in 100 mL normal saline, administered once through intravenous infusion over a 40-minute duration. The placebo infusion was normal saline (0.9% sodium chloride 100 mL). For Stage 2, the experimental drug was a fixed-dose combination of DCS and lurasidone (NRX-101; NRx Pharmaceuticals, Wilmington, DE) and the comparator was lurasidone at the same dosage as in the matched experimental drug. NRX-101 and lurasidone comparator were manufactured to Good Manufacturing Practices standards at an FDA-inspected facility (WuXi Apptec; Shanghai, CN). NRX-101 was administered orally twice a day, starting on Day 1 or 2 depending on the timing of the response in Stage 1, and continued for six weeks. The total doses of DCS and lurasidone were 350 mg and 16.5 mg, respectively, for the first administration and were uptitrated to 950 mg DCS and 66 mg lurasidone by the fifth administration. After clinical evaluation, the blinded Investigator could up-titrate the lurasidone dose two steps (to 99 and 132 mg/day) or down-titrate DCS and lurasidone two steps (DCS: 825 and 700 mg/day; lurasidone 49.5 and 33 mg/day). Due to reports from the tuberculosis literature suggesting that high-dose DCS may alter pyridoxine, all patients were given vitamin B6 tablets (pyridoxine 100 mg) and instructed to take one tablet daily.

### Randomization

Stage 1 randomization at study entry assigned patients to ketamine versus saline in a 3:1 ratio. Stage 2 randomization assigned patients to DCS plus lurasidone versus lurasidone in a 2:1 ratio. Stage 1 randomization was stratified by baseline CSSRS score (4 vs. 5), presence vs. absence of sub-syndromal hypomanic symptoms (BISS mania score), and evidence of a suicidal event in the prior 12 months ([including an attempt, an emergency department visit, or hospitalization for suicidality or mental health encounter prompted by suicidality). Baseline demographics and stratification factors are included in Appendix 1. All patients with at least a minimal improvement (defined as ≥25% improvement in BDM, and C-SSRS ≤ 3) assessed 24 and 48 h after infusion were offered the option to continue into Stage 2, a 42-day treatment stage.

Stage 2 randomization was stratified by the Stage 1 treatment, by the Stage 1 response [partial (25–49% CFB BDM) or full response (≥ 50%)], and if directly assigned to ketamine (if saline infusion was declared futile by the Data Safety Monitoring Board). IWRS failures requiring manual treatment assignments were recorded. Adherence to oral doses of investigational product was documented via the AiCure telemedicine system, which were referenced against blood levels of investigational drug pharmacokinetic (PK) data.

### Outcome measures

The primary efficacy endpoint for the Stage 1 was the proportion of patients with a successful response, as measured by acutely diminished suicidality and improved mood (achieving C-SSRS ≤ 3 and an acute ≥ 25% improvement in the BDM) within 48 h. The primary efficacy endpoint for Stage 2 was the improvement in the BDM from Stage 2 baseline through Day 42 of the treatment in those patients who responded in Stage 1.

The key secondary efficacy endpoint of Stage 1 was the difference in mean change on the BDM between ketamine and saline groups assessed at Day 1 and Day 2. The planned key secondary efficacy endpoint in Stage 2 was the difference in time to relapse between the two treatment arms. Relapse was defined as experiencing a 25% or greater return to pre-ketamine baseline levels of depression, or suicidality, or the need to implement a new treatment plan. Because no instances of relapse (adjudicated by a three-physician Relapse Adjudication Committee) occurred in the NRX-101 group, a time-to-event analysis could not be performed and relapse rates of the two treatment groups were compared.

Akathisia is a known side effect of medications that inhibit the 5-HT_2A_ receptor and is potentially associated with increased suicidal ideation and behavior (Akagi and Kumar 2002). Accordingly, the Barnes Akathisia Rating Scale (BARS) was included as a safety measure (Barnes 1989).

### Pharmacokinetics measurements

Blood samples for PK analysis of ketamine were collected one hour prior to and two hours after the initial administration. Blood samples for monitoring plasma DCS and lurasidone levels were collected from all patients to verify exposure and consistency with randomization assignment. Samples for the trough determination were drawn within one hour before the drug administration on Days 14 and 42, whereas samples for the steady state Cmax determination were drawn two hours ± 15 min after the drug administration on Day 42. Additional PK samples were obtained from patients with serious adverse events (SAEs) to characterize potential relationships between AEs and plasma levels. An unblinded medical monitor reviewed these data on an ongoing basis.

### Statistical methods

All analyses were performed using SAS statistical software, version 9.4 or later. This study was originally powered at Stage 1 (n = 140) to detect a 30% difference in postinfusion response rates and powered at Stage 2 (n = 72) to detect a 40% reduction in relapse rates. Because the study was stopped early to accommodate the Special Protocol Agreement with FDA, 20 patients were planned to be enrolled to confirm protocol feasibility and drug exposure. However, 22 patients were enrolled because two additional patients had consented when study termination was announced. The study, as enrolled, had 80% power to detect a seven-point mean difference in primary endpoint. This magnitude of difference was observed in prior trials (Crane 1959).

For the primary endpoints, descriptive statistics and Mixed Model Repeated Measures (MMRM)-based average scores and pvalues were reported by visit and overall. The primary analysis was based on Last Observation Carried Forward (LOCF), as is typical in antidepressant trials (Loebel et al. 2014). The proportion of responders and relapses between the two treatments was compared using a two-sided Fisher Exact Test. Three rating scales—Brief Psychiatric Scale (BPRS+), the Clinical Global Impression of Severity for Suicide (CGI-SS), and C-SSRS—were analyzed using paired t-tests on within treatment change and unpaired t-tests to compare change from baseline differences between treatments. All rating sessions were captured digitally and transmitted to experienced central master raters.

## Results

### Baseline characteristics and Disposition of patients

**Fig. 1:**
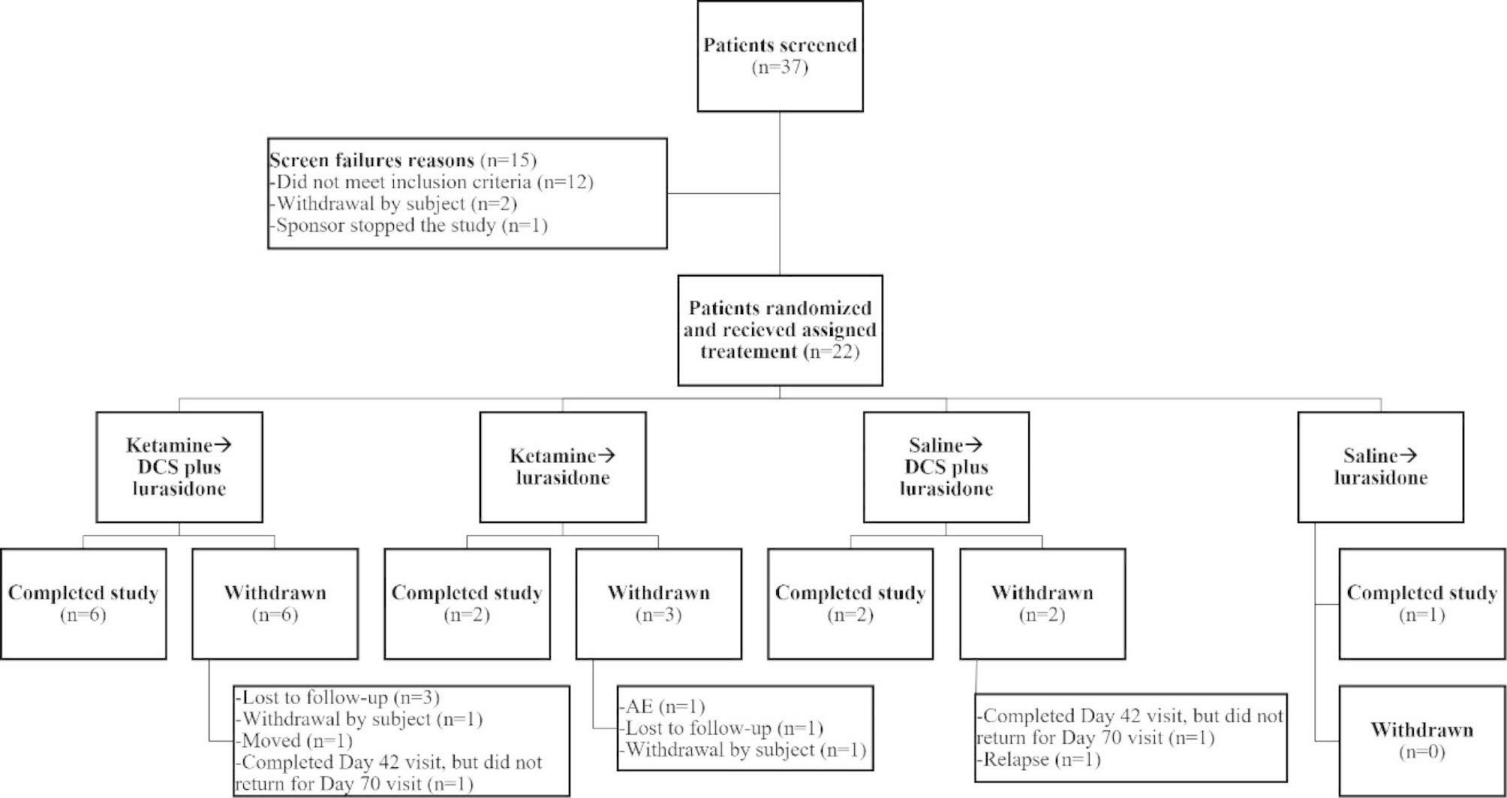
CONSORT Diagram of Screened and Enrolled Participants

Thirty-seven patients were screened in this study, 22 of whom were eligible for enrolment. All 22 patients were randomized (Fig. 1) and received either ketamine (n = 17) or saline (n = 5) in Stage (1) All 22 patients who were randomized to receive a study treatment were included in the safety population and analysis population (i.e., intent-to-treat) and all received lurasidone with or without DCS. The mean age of patients was 39.5 years (Table 1). Of the 17 patients who received ketamine, 12 patients were subsequently randomized to oral NRX-101 (ketamine → NRX-101) and five were subsequently randomized to lurasidone (ketamine → lurasidone) in Stage (2) Eleven patients (50%) discontinued from the study. No patients were lost to follow-up.

Twenty-one of the 22 patients presented with a baseline score ≥ 4 on the C-SSRS [ketamine, n = 16 (94.1%); Saline, n = 5 (100%)]. One subject presented at study intake with C-SSRS = 5 but was noted to have C-SSRS = 1 at the pre-infusion assessment 24 h later and did not progress to stage 2. Most patients [n = 17 (77.3%)] did not present with hypomanic symptoms on the BISS [ketamine, n = 13 (76.5%); Saline, n = 4 (80.0%)]. Eighteen of the 22 patients had experienced at least one suicidal event in the past 12 months [ketamine, n = 14 (82.4%); saline, n = 4 (80.0%)] (Table 1).

**Table 1:** Baseline Characteristics and Stratification Factors

Parameter	Result	Saline (N = 5)	Ketamine (N = 17)	ketamine		saline		All Patients (N = 22)
				DCS plus lurasidone (N = 12)	lurasidone (N = 5)	DCS plus lurasidone (N = 4)	lurasidone (N = 1)	
Age	Mean	43.6	38.4	33.9	49.0	41.0	54.0	39.5
	Median	46.0	42.0	30.0	45.0	42.0	54.0	42.5
	Std Dev	9.63	11.90	10.47	8.03	8.87	0	11.43
	Min, Max	30, 54	19, 60	19, 48	42, 60	30, 50	54, 54	19, 60
	95% CI	(31.6, 55.6)	(32.2, 44.5)	(27.3, 40.6)	(39.0, 59.0)	(26.9, 55.1)	0	(34.5, 44.6)
Sex [n (%)]	Male	3 (60.0)	13 (76.5)	9 (75.0)	4 (80.0)	2 (50.0)	1 (100)	16 (72.7)
	Female	2 (40.0)	4 (23.5)	3 (25.0)	1 (20.0)	2 (50.0)	0	6 (27.3)
Race [n (%)]	White	3 (60.0)	16 (94.1)	11 (91.7)	5 (100)	3 (75.0)	0	19 (86.4)
	Black or African-American	2 (40.0)	1 (5.9)	1 (8.3)	0	1 (25.0)	1 (100)	3 (13.6)
Ethnicity [n (%)]	Hispanic	0	0	0	0	0	0	0
	Non-Hispanic	5 (100)	17 (100)	12 (100)	5 (100)	4 (100)	1 (100)	22 (100)
Baseline C-SSRS	1	0	1 (5.9)	1 (8.3)	0	0	0	1 (4.5)
Score [n (%)]*	4	0	2 (11.8)	2 (16.7)	0	0	0	2 (9.1)
	5	5 (100)	14 8 (2.4)	9 (75.0)	5 (100)	4 (100)	1 (100)	19 (86.4)
Hypomanic	None	4 (80.0)	13 (76.5)	9 (75.0)	4 (80.0)	3 (75.0)	1 (100)	17 (77.3)
symptoms (BISS)	Any	1 (20.0)	4 (23.5)	3 (25.0)	1 (20.0)	1 (25.0)	0	5 (22.7)
Suicidal Event in	None	1 (20.0)	3 (17.6)	3 (25.0)	0	1 (25.0)	0	4 (18.2)
Prior 12 Months	Any	4 (80.0)	14 (82.4)	9 (75.0)	5 (100)	3 (75.0)	1 (100)	18 (81.8)

BISS = Bipolar Inventory of Symptoms Scale; C-SSRS = Columbia Suicide Severity Rating Scale

* One participant achieved spontaneous remission from suicidality prior to ketamine infusion and did not progress to stage 2 randomization

### Efficacy results

**Fig. 2:**
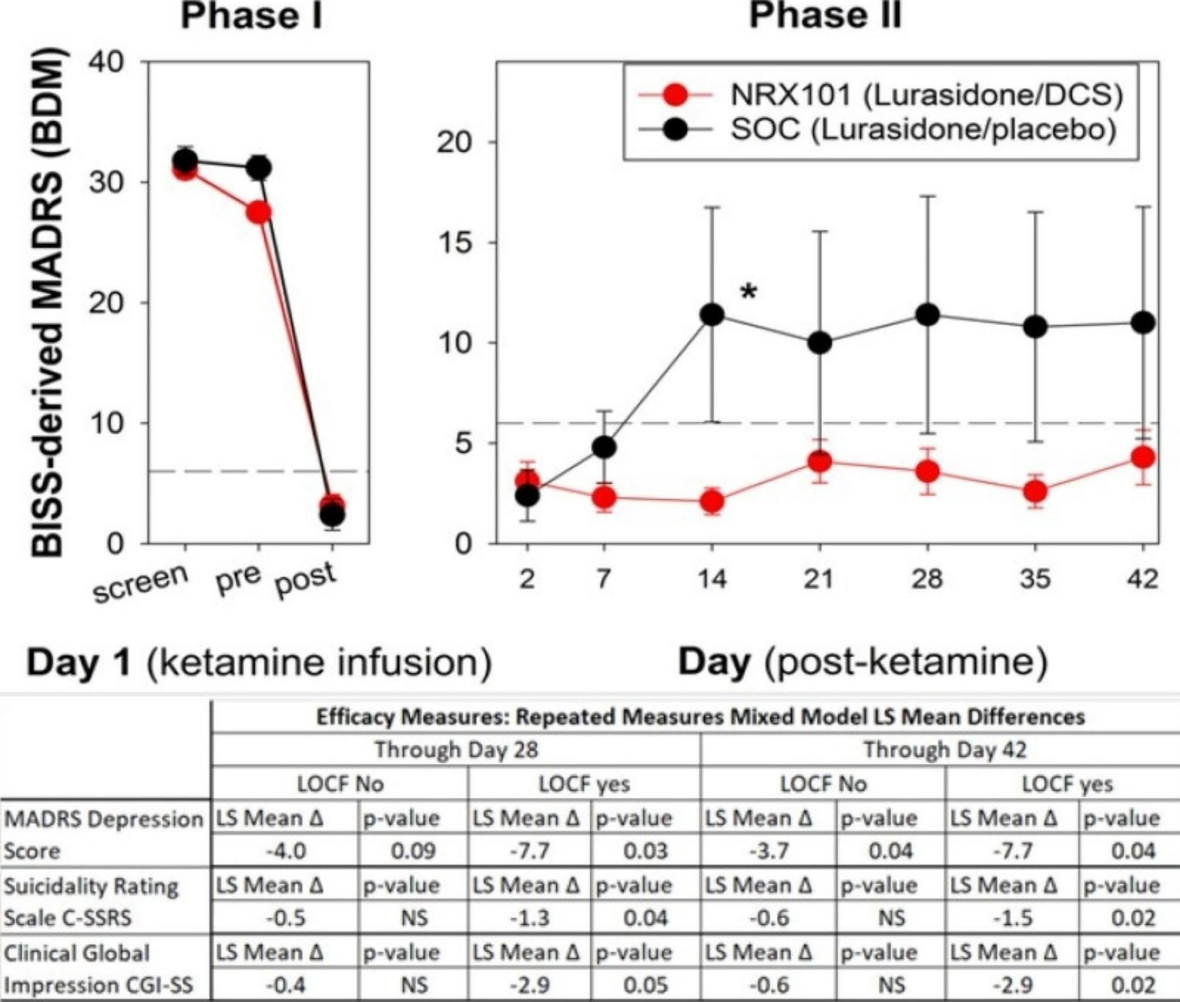
(Graph) Primary Endpoint for those infused with ketamine vs. placebo in phase I (n=22) and those who responded to ketamine in phase I and were randomized in phase II to receive either NRX-101 or lurasidone (n=17). Table depicts primary and secondary endpoints for those randomized in phase II (n=17).; *, P<0.05

The Stage 1 efficacy analyses demonstrated that both ketamine (n = 17) and saline (n = 5) were associated with a significant reduction in suicidal ideation at 24- and 48-hours post-infusion (primary efficacy endpoint; Fig. 2) and improved BDM, CGI-SS, and C-SSRS from pre-infusion baseline in the intention to treat cohort. There were no obvious differences between ketamine and saline treatment groups. Thus, the 48-hour period of hospital supervision was associated with remission from depression and suicidality in all subjects.

Primary and secondary endpoints for stage 2 are illustrated in Fig. 2. For the Stage 2 ITT population, the primary outcome measure was change in BDM between baseline and Day 42. MMRM regression showed that ketamine → NRX-101 treatment was associated with significantly lower BDM scores compared to ketamine → lurasidone treatment through Days 28 and 42 (p = 0.0402 and p = 0.0324, respectively) after LOCF analysis. Without LOCF, the difference was significant at Day 28 (p = 0.0425) and consistent at Day 42 (p = 0.0908). No differences were observed when stratifying for Stage 1 treatment. The 7.7 mean difference between treatments for the change from baseline to day 42 had an effect size of 1.19 (95% CI: 0.38, 2.00). The effect size for CGI suicidality was 1.38 (95% CI: 0.27, 2.49), which was comparable.

**Table 2:** Stage 2 BDM Response Profile Mixed Model Repeated Measures

Populations	Day	lurasidone (N = 5)	NRX-101 (N = 12)	NRX-101 - lurasidone (N = 17)
		LSMean	LSMean	LSDifference (p-value)
No LOCF	2	2.4	3.0	0.6 (0.83)
	7	4.6	1.7	-2.9 (0.36)
	14	12.9	2.0	-10.9 (0.002)
	21	5.7	5.5	-0.2 (0.95)
	28	7.7	4.0	-3.7 (0.30)
	BDM 10 Response Profile p-value			0.042
	35	7.3	3.3	-4.0 (0.29)
	42	7.7	4.9	-2.8 (0.46)
	BDM 10 Response Profile p-value			0.09
LOCF	2	2.4	3.0	0.6 (0.85)
	7	4.8	2.0	-2.8 (0.39)
	14	11.4	2.2	-9.2 (0.006)
	21	10.0	4.5	-5.5 (0.09)
	28	11.2	3.5	-7.7 (0.02)
	BDM 10 Response Profile p-value			0.04
	35	10.8	2.8	-8.0 (0.02)
	42	11.0	3.7	-7.3 (0.04)
	BDM 10 Response Profile p-value			0.03

BDM = BISS-derived MADRS score; ITT1 = Intent-to-Treat Stage 1 Population; ITT2 = Intent-to-Treat Stage 2 Population; LOCF = Last Observation Carried Forward

The key secondary efficacy endpoint for Stage 1 was the difference in mean change in the BDM from pre-infusion baseline to final observation (Day 1 or Day 2 post-infusion) between ketamine and saline treatment groups. For the Stage 1 ITT population, the mean baseline BDM score reported at pre-infusion was 29.6 (range: 12–38; ketamine, mean: 28.9, range: 12–38; saline, mean: 31.8, range: 29–37). Both ketamine and saline treatment statistically significantly decreased the mean BDM score at Visit 1 (ketamine, mean: 2.6, range: 0–8, p < 0.0001; saline, mean: 5.6, range: 0–19, p = 0.0033) and at the end of Stage 1 (ketamine, mean 3.3, range: 0–9, p < 0.0001; saline, 5.6, range: 0–19, p = 0.0033). There were no statistically significant differences in the mean BDM score between ketamine and saline treatment groups. Neither ketamine nor saline treatment significantly changed the mean BPRS + score.

The updated key secondary efficacy endpoint for Stage 2 was the difference in the relapse rate between DCS plus lurasidone and lurasidone treatment groups. There was no significant difference observed after stratifying the Stage 2 ITT population by its Stage 1 saline treatment (Saline → DCS plus lurasidone and Saline → lurasidone, p = 1.000). There was, however, a trend towards a decrease in the relapse rate of the ketamine → NRX-101 group (0%, 0/12 patients) compared to that of the ketamine → lurasidone group (40%, 2/5 patients) (p = 0.0735).

The Clinical Global Impression – Suicidality Scale (CGI-SS) was both a secondary efficacy measure and a safety measure in this study. The Stage 1 pre-infusion baseline mean CGI-SS total score of the ITT population was 17.4 (range: 10–20; ketamine, mean: 17.0, range: 10–20; saline, mean: 18.6, range: 14–20). Both ketamine and saline treatment statistically significantly decreased the mean CGI-SS score from pre-infusion baseline to Visit 1 (ketamine, mean: 2.1, range: 0–9, p < 0.0001; saline mean: 1.2, range: 0–4, p = 0.0009) and End of Stage 1 (ketamine, mean: 2.1, range: 0–9, p < 0.0001; saline, mean: 1.2, range: 0–4, p = 0.0009). There were no statistically significant differences observed between ketamine- and saline-treated patients.

MMRM regression was used to interpret changes in CGI-SS for Stage 2 data both without and with LOCF. For the Stage 2 ITT population, ketamine → NRX-101 treatment significantly lowered CGI-SS Response Profiles compared to ketamine → lurasidone treatment through Day 28 and Day 42 when imputing LOCF (p = 0.0457 and p = 0.0186, respectively), but not without LOCF (p = 0.1485 and p = 0.1527, respectively). No differences were observed in the CGI-SS Response Profiles of MMRM by Stage 1 treatment through Days 28 and 42. With LOCF analyzed, these data suggest that ketamine followed by NRX-101 treatment is significantly associated with a lower (i.e., better) rating of the subject’s suicidality by the treating physician compared to ketamine → lurasidone treatment.

The pre-infusion baseline mean C-SSRS score (suicidal ideation) for the Stage 1 ITT population was 4.7 (range: 1–5; ketamine, mean: 4.6, range: 1–5; saline, mean: 5.0, range: 5–5). Both ketamine and saline treatment statistically significantly decreased the mean CSSRS scores from pre-infusion baseline to Visit 1–4 h, Visit 1–10 h, Visit 1–24 h, and End of Stage 1. There was no statistically significant difference observed between ketamine and saline treatment groups.

For Stage 2, MMRM regression was performed first without LOCF then repeated with LOCF. For the ITT population, ketamine → NRX-101 treatment significantly lowered C-SSRS (ideation) Response Profiles compared to ketamine → lurasidone treatment through Day 28 and Day 42 when imputing LOCF (p = 0.0419 and p = 0.0210, respectively) but not without LOCF through Days 28 and 42 (p = 0.1125 and p = 0.1112, respectively). No difference was observed in the C-SSRS Response Profiles after stratifying by Stage 1 treatment through Days 28 and 42.

### Safety results

In the Stage 1 Safety population, 11 of the 17 ketamine-treated patients (64.7%) experienced a total of 29 AEs whereas no subject treated with saline (0%) experienced an AE (Supplementary Table 1). None of these 29 AEs was considered severe and no SAE was recorded. Eleven (68.8%) DCS plus lurasidone-treated patients in the Stage 2 Safety population experienced 36 total AEs, none of which was considered severe and no SAE was experienced.

Overall, no severe AEs, SAEs, or deaths were experienced in patients treated with ketamine → NRX-101. Three subjects in the ketamine → lurasidone group experienced SAEs (angina pectoris, suicidal ideation, and wound). Detailed safety information is provided in Appendix 3.

The trial was not powered to prove a statistically significant difference on BARS akathisia scores. However, the study demonstrates a 1.0-point increase from baseline in the lurasidone group and a 0.2-point decrease in BARS score in the NRX-101 group (d = 1.1; t-test on difference; p = 0.14). This trend is consistent with the preclinical data and suggests a meaningful effect in reduction of lurasidone-induced akathisia might be seen in an adequately powered trial.

### Pharmacokinetics results

DCS and lurasidone blood concentrations were recorded for the Stage 2 Safety populations. The blood levels obtained were consistent with oral ingestion of the investigational product. Detailed PK methods and data are shown in Appendix 2.

## Discussion

The results of this Phase 2 study in 22 patients demonstrate that a sequential treatment of a single infusion of ketamine followed by NRX-101, a fixed-dose combination of DCS and lurasidone, is significantly more efficacious than ketamine followed by lurasidone at maintaining reduction in depression (p = 0.03) and suicidal ideation, as measured by C-SSRS (p = 0.02) and by CGI-SS (p = 0.03). There is a trend toward a reduction in relapse (p = 0.07). However, ketamine treatment alone did not demonstrate superiority in treating acute suicidal ideation and behavior compared to saline, likely because of the substantial ketamine expectation effect that is now well known in the literature.

The statistically significant separation in Stage 2 between NRX-101 and lurasidone alone remained significant on MMRM throughout the 42-day time interval. These findings suggest that the addition of DCS achieved a superior effect compared to lurasidone alone in maintaining both the antidepressant effect and the reduction in suicidal ideation. The decrease in suicidal ideation observed with NRX-101 was seen both on C-SSRS and MADRS as assessed by independent raters and on the CGI-SS scale ascertained by the treating site physician, suggesting a consistency in effect. This beneficial effect on suicidality associated with DCS was also reported by Chen (Chen et al. 2019) in patients with bipolar disorder who were actively suicidal at baseline.

While the sample size in this study was not sufficient to prove a statistically significant difference in relapse (0/12 v. 2/5; p = 0.07), the observed numerical difference suggests that a meaningful difference may be seen in an adequately powered trial. Patients in this study were released from the hospital within two days and required hospitalization only after relapse.

No significant safety events were observed following infusion of either ketamine or saline placebo. No SAEs occurred in the ketamine → NRX-101 group. However, three SAEs were reported in the ketamine → lurasidone group with two instances of hospitalization for relapse. Therefore, the sequential regimen of a single infusion of ketamine followed by NRX-101 appears to be safe well tolerated.

Nonclinical studies have found a significant reduction in akathisia when DCS and other NMDAR antagonists are added to lurasidone and other D_2_/5-HT_2A_ antagonists. While this trial was underpowered to prove a difference in akathisia between the NRX-101 and lurasidone only groups, a high effect size was seen (d = 1.1) with an increase in akathisia seen in the lurasidone group and a decrease in akathisia seen in the NRX-101 cohort at a trend level of significance (p = 0.14). This effect will be further explored in future trials.

Based on these findings, the FDA awarded Breakthrough Therapy Designation and granted a Special Protocol Agreement for a Phase 3 registration trial of NRX-101 vs. lurasidone in treatment of patients with Severe Bipolar Depression and Acute Suicidal Ideation or Behavior (NCT03396068).

## Conclusion

Patients treated with an oral NMDAR antagonist medication (D-cycloserine) as part of a fixed-dose combination with lurasidone (NRX-101), a third-generation antipsychotic indicated for treatment of bipolar depression following improvement with ketamine, were shown to have significantly lower levels of depression and ASIB, together with a numerical trend towards reduced likelihood of relapse and rehospitalization, than were patients treated with lurasidone alone. A numerical trend towards reduced levels of akathisia was seen (p = 0.14) in the NRX-101-treated patients, consistent with findings in non-clinical studies. Registration studies under an FDA Special Protocol Agreement and Breakthrough Therapy Designation are now underway.

### Electronic supplementary material

Below is the link to the electronic supplementary material.

Supplementary Material 1

## Data Availability

The datasets used and/or analyzed during the current study are available from the corresponding author on reasonable request.
