# Supplementary material for: NRX-101 (D-cycloserine plus lurasidone) vs. lurasidone for the maintenance of initial stabilization after ketamine in patients with severe bipolar depression with acute suicidal ideation and behavior: a randomized prospective phase 2 trial
Source: Int J Bipolar Disord. 2023 Aug 13;11:28. doi: 10.1186/s40345-023-00308-5 (PMC10423711; doi:10.1186/s40345-023-00308-5)
Supplement: Supplementary file 1 — Supplementary Material 1 [file 40345_2023_308_MOESM1_ESM.docx]

**Supplementary Materials**

**Appendix 1**

**Additional Demographic Data**

**Stage 1 ITT1 Population**

|  | | NRX-100 | | Saline | |  |
| --- | --- | --- | --- | --- | --- | --- |
| Parameter | Result | NRX-101  (N=10) | lurasidone  (N=5) | NRX-101  (N=4) | lurasidone  (N=1) | All Subjects  (N=20) |
| Age | N | 10 | 5 | 4 | 1 | 20 |
|  | Mean | 32.4 | 49.0 | 41.0 | 54.0 | 39.4 |
|  | Median | 28.5 | 45.0 | 42.0 | 54.0 | 42.5 |
|  | Std Dev | 10.57 | 8.03 | 8.87 |  | 11.86 |
|  | Min, Max | 19, 48 | 42, 60 | 30, 50 | 54, 54 | 19, 60 |
|  | 95% CI | (24.8, 40.0) | (39.0, 59.0) | (26.9, 55.1) |  | (33.8, 44.9) |
|  |  |  |  |  |  |  |
| Sex [n (%)] | Male | 8 (80.0) | 4 (80.0) | 2 (50.0) | 1 (100) | 15 (75.0) |
|  | Female | 2 (20.0) | 1 (20.0) | 2 (50.0) | 0 | 5 (25.0) |
|  |  |  |  |  |  |  |
| Race [n (%)] | White | 10 (100) | 5 (100) | 3 (75.0) | 0 | 18 (90.0) |
|  | Black or African-American | 0 | 0 | 1 (25.0) | 1 (100) | 2 (10.0) |
|  | Asian | 0 | 0 | 0 | 0 | 0 |
|  | American Indian or Alaskan Native | 0 | 0 | 0 | 0 | 0 |
|  | Native Hawaiian or Other Pacific Islander | 0 | 0 | 0 | 0 | 0 |
|  | Other (including multiple) | 0 | 0 | 0 | 0 | 0 |
|  |  |  |  |  |  |  |
| Ethnicity [n (%)] | Hispanic | 0 | 0 | 0 | 0 | 0 |
|  | Non-hispanic | 10 (100) | 5 (100) | 4 (100) | 1 (100) | 20 (100) |
|  |  |  |  |  |  |  |
|  | | NRX-100 | | Saline | |  |
| Parameter | Result | NRX-101  (N=10) | lurasidone  (N=5) | NRX-101  (N=4) | lurasidone  (N=1) | All Subjects  (N=20) |
| Baseline C-SSRS Score [n (%)] | 1 | 1 (10.0) | 0 | 0 | 0 | 1 (5.0) |
|  | 4 | 2 (20.0) | 0 | 0 | 0 | 2 (10.0) |
|  | 5 | 7 (70.0) | 5 (100) | 4 (100) | 1 (100) | 17 (85.0) |
|  |  |  |  |  |  |  |
| Hypomanic symptoms by BISS [n (%)] | None | 7 (70.0) | 4 (80.0) | 3 (75.0) | 1 (100) | 15 (75.0) |
|  | Any | 3 (30.0) | 1 (20.0) | 1 (25.0) | 0 | 5 (25.0) |
|  |  |  |  |  |  |  |
| Suicidal Event in Prior 12 Months [n (%)] | None | 3 (30.0) | 0 | 1 (25.0) | 0 | 4 (20.0) |
|  | Any | 7 (70.0) | 5 (100) | 3 (75.0) | 1 (100) | 16 (80.0) |
|  |  |  |  |  |  |  |
| Screening MADRS(10) Score | N | 10 | 5 | 4 | 1 | 20 |
|  | Mean | 31.1 | 31.8 | 33.5 | 33.0 | 31.9 |
|  | Median | 30.5 | 31.0 | 33.5 | 33.0 | 31.0 |
|  | Std Dev | 2.73 | 2.59 | 4.20 |  | 2.94 |
|  | Min, Max | 26, 35 | 29, 36 | 29, 38 | 33, 33 | 26, 38 |
|  | 95% CI | (29.1, 33.1) | (28.6, 35.0) | (26.8, 40.2) |  | (30.5, 33.2) |
|  |  |  |  |  |  |  |

**Stage 1 Safety Population**

|  | | NRX-100 | | Saline | |  |
| --- | --- | --- | --- | --- | --- | --- |
| Parameter | Result | NRX-101  (N=12) | lurasidone  (N=5) | NRX-101  (N=4) | lurasidone  (N=1) | All Subjects  (N=22) |
| Age | N | 12 | 5 | 4 | 1 | 22 |
|  | Mean | 33.9 | 49.0 | 41.0 | 54.0 | 39.5 |
|  | Median | 30.0 | 45.0 | 42.0 | 54.0 | 42.5 |
|  | Std Dev | 10.47 | 8.03 | 8.87 |  | 11.43 |
|  | Min, Max | 19, 48 | 42, 60 | 30, 50 | 54, 54 | 19, 60 |
|  | 95% CI | (27.3, 40.6) | (39.0, 59.0) | (26.9, 55.1) |  | (34.5, 44.6) |
|  |  |  |  |  |  |  |
| Sex [n (%)] | Male | 9 (75.0) | 4 (80.0) | 2 (50.0) | 1 (100) | 16 (72.7) |
|  | Female | 3 (25.0) | 1 (20.0) | 2 (50.0) | 0 | 6 (27.3) |
|  |  |  |  |  |  |  |
| Race [n (%)] | White | 11 (91.7) | 5 (100) | 3 (75.0) | 0 | 19 (86.4) |
|  | Black or African-American | 1 (8.3) | 0 | 1 (25.0) | 1 (100) | 3 (13.6) |
|  | Asian | 0 | 0 | 0 | 0 | 0 |
|  | American Indian or Alaskan Native | 0 | 0 | 0 | 0 | 0 |
|  | Native Hawaiian or Other Pacific Islander | 0 | 0 | 0 | 0 | 0 |
|  | Other (including multiple) | 0 | 0 | 0 | 0 | 0 |
|  |  |  |  |  |  |  |
| Ethnicity [n (%)] | Hispanic | 0 | 0 | 0 | 0 | 0 |
|  | Non-hispanic | 12 (100) | 5 (100) | 4 (100) | 1 (100) | 22 (100) |
|  |  |  |  |  |  |  |
|  | | NRX-100 | | Saline | |  |
| Parameter | Result | NRX-101  (N=12) | lurasidone  (N=5) | NRX-101  (N=4) | lurasidone  (N=1) | All Subjects  (N=22) |
| Baseline C-SSRS Score [n (%)] | 1 | 1 (8.3) | 0 | 0 | 0 | 1 (4.5) |
|  | 4 | 2 (16.7) | 0 | 0 | 0 | 2 (9.1) |
|  | 5 | 9 (75.0) | 5 (100) | 4 (100) | 1 (100) | 19 (86.4) |
|  |  |  |  |  |  |  |
| Hypomanic symptoms by BISS [n (%)] | None | 9 (75.0) | 4 (80.0) | 3 (75.0) | 1 (100) | 17 (77.3) |
|  | Any | 3 (25.0) | 1 (20.0) | 1 (25.0) | 0 | 5 (22.7) |
|  |  |  |  |  |  |  |
| Suicidal Event in Prior 12 Months [n (%)] | None | 3 (25.0) | 0 | 1 (25.0) | 0 | 4 (18.2) |
|  | Any | 9 (75.0) | 5 (100) | 3 (75.0) | 1 (100) | 18 (81.8) |
|  |  |  |  |  |  |  |
| Screening MADRS(10) Score | N | 12 | 5 | 4 | 1 | 22 |
|  | Mean | 31.9 | 31.8 | 33.5 | 33.0 | 32.2 |
|  | Median | 31.5 | 31.0 | 33.5 | 33.0 | 31.5 |
|  | Std Dev | 3.12 | 2.59 | 4.20 |  | 3.05 |
|  | Min, Max | 26, 36 | 29, 36 | 29, 38 | 33, 33 | 26, 38 |
|  | 95% CI | (29.9, 33.9) | (28.6, 35.0) | (26.8, 40.2) |  | (30.9, 33.6) |
|  |  |  |  |  |  |  |

**Appendix 2**

**Pharmacokinetics Results**

DCS and lurasidone HCl blood concentrations were recorded for the Stage 2 Safety populations (supplemental Table 5). DCS and lurasidone were not detectable before the drug administration. For patients treated with NRX-101 in the Safety population, the mean DCS concentration was 5.709 µg/mL, 17.820 µg/mL, 12.104 µg/mL, and 24.373 µg/mL at Visit 2 post-dose, Visit 7 post-dose, Visit 11 pre-dose, and Visit 11 post-dose, respectively, and the mean lurasidone concentration was 10.366 ng/mL, 10.135 ng/mL, 10.859 ng/mL, and 36.965 ng/mL at Visit 2 post-dose, Visit 7 post-dose, Visit 11 pre-dose, and Visit 11 post-dose, respectively (Table 5). For patients treated with lurasidone, the mean lurasidone concentration was 9.813 ng/mL, 9.688 ng/mL, 7.227 ng/mL, and 27.750 ng/mL at Visit 2 post-dose, Visit 7 post-dose, Visit 11 pre-dose, and Visit 11 post-dose, respectively (Table 5).

**Table 1 - Summary of Treatment Emergent Adverse Events: Stage 1 Safety Population**

|  | Saline  (N=5) | | Ketamine (N=17) | | Total  (N=22) | |
| --- | --- | --- | --- | --- | --- | --- |
| SYSTEM ORGAN CLASS     Preferred Term | N (%) of  Subjects | N of  Events | N (%) of  Subjects | N of  Events | N (%) of  Subjects | N of  Events |
| Total Subjects with Any AE | 0 | 0 | 11 (64.7) | 29 | 11 (50.0) | 29 |
| EAR AND LABYRINTH DISORDERS | 0 | 0 | 3 (17.6) | 3 | 3 (13.6) | 3 |
| Tinnitus | 0 | 0 | 3 (17.6) | 3 | 3 (13.6) | 3 |
| EYE DISORDERS | 0 | 0 | 2 (11.8) | 3 | 2 (9.1) | 3 |
| Diplopia | 0 | 0 | 1 (5.9) | 1 | 1 (4.5) | 1 |
| Blurred vision | 0 | 0 | 2 (11.8) | 2 | 2 (9.1) | 2 |
| GASTROINTESTINAL DISORDERS | 0 | 0 | 4 (23.5) | 4 | 4 (18.2) | 4 |
| Diarrhea | 0 | 0 | 1 (5.9) | 1 | 1 (4.5) | 1 |
| Dry mouth | 0 | 0 | 1 (5.9) | 1 | 1 (4.5) | 1 |
| Nausea | 0 | 0 | 1 (5.9) | 1 | 1 (4.5) | 1 |
| Vomiting | 0 | 0 | 1 (5.9) | 1 | 1 (4.5) | 1 |
| NERVOUS SYSTEM DISORDERS | 0 | 0 | 5 (29.4) | 8 | 5 (22.7) | 8 |
| Coordination abnormal | 0 | 0 | 1 (5.9) | 1 | 1 (4.5) | 1 |
| Dizziness | 0 | 0 | 2 (11.8) | 3 | 2 (9.1) | 3 |
| Dysmetropsia | 0 | 0 | 1 (5.9) | 1 | 1 (4.5) | 1 |
| Headache | 0 | 0 | 1 (5.9) | 1 | 1 (4.5) | 1 |
| Hypoesthesia | 0 | 0 | 2 (11.8) | 2 | 2 (9.1) | 2 |
| PSYCHIATRIC DISORDERS | 0 | 0 | 3 (17.6) | 6 | 3 (13.6) | 6 |
| Dissociation | 0 | 0 | 2 (11.8) | 2 | 2 (9.1) | 2 |
| Euphoric mood | 0 | 0 | 2 (11.8) | 2 | 2 (9.1) | 2 |
| Restlessness | 0 | 0 | 2 (11.8) | 2 | 2 (9.1) | 2 |
| SKIN AND SUBCUTANEOUS TISSUE DISORDERS | 0 | 0 | 2 (11.8) | 2 | 2 (9.1) | 2 |
| Dry skin | 0 | 0 | 1 (5.9) | 1 | 1 (4.5) | 1 |
| Rash | 0 | 0 | 1 (5.9) | 1 | 1 (4.5) | 1 |
| VASCULAR DISORDERS | 0 | 0 | 3 (17.6) | 3 | 3 (13.6) | 3 |
| Hypertension | 0 | 0 | 3 (17.6) | 3 | 3 (13.6) | 3 |

AE = Adverse Event

**Table 2 - Summary of Treatment Emergent Adverse Events: Stage 2 Safety Population**

|  | Lurasidone HCl  (N=6) | | NRX-101  (N=16) | | Total  (N=22) | |
| --- | --- | --- | --- | --- | --- | --- |
| SYSTEM ORGAN CLASS     Preferred Term | N (%) of  Subjects | N of  Events | N (%) of  Subjects | N of  Events | N (%) of  Subjects | N of  Events |
| Total Subjects with Any AE | 4 (66.7) | 11 | 11 (68.8) | 36 | 15 (68.2) | 47 |
| CARDIAC DISORDERS | 1 (16.7) | 1 | 1 (6.3) | 1 | 2 (9.1) | 2 |
| Angina pectoris | 1 (16.7) | 1 | 0 | 0 | 1 (4.5) | 1 |
| Palpitations | 0 | 0 | 1 (6.3) | 1 | 1 (4.5) | 1 |
| MUSCULOSKELETAL AND CONNECTIVE TISSUE DISORDERS | 0 | 0 | 1 (6.3) | 1 | 1 (4.5) | 1 |
| Muscle spasms | 0 | 0 | 1 (6.3) | 1 | 1 (4.5) | 1 |
| NERVOUS SYSTEM DISORDERS | 1 (16.7) | 1 | 6 (37.5) | 9 | 7 (31.8) | 10 |
| Akathisia | 0 | 0 | 1 (6.3) | 3 | 1 (4.5) | 3 |
| Headache | 0 | 0 | 1 (6.3) | 1 | 1 (4.5) | 1 |
| Hypersomnia | 0 | 0 | 1 (6.3) | 1 | 1 (4.5) | 1 |
| Lethargy | 0 | 0 | 1 (6.3) | 1 | 1 (4.5) | 1 |
| Sedation | 1 (16.7) | 1 | 2 (12.5) | 2 | 3 (13.6) | 3 |
| Somnolence | 0 | 0 | 1 (6.3) | 1 | 1 (4.5) | 1 |
| PSYCHIATRIC DISORDERS | 3 (50.0) | 4 | 5 (31.3) | 8 | 8 (36.4) | 12 |
| Anorgasmia | 0 | 0 | 1 (6.3) | 1 | 1 (4.5) | 1 |
| Depressed mood | 0 | 0 | 1 (6.3) | 1 | 1 (4.5) | 1 |
| Depression | 1 (16.7) | 1 | 2 (12.5) | 2 | 3 (13.6) | 3 |
| Restlessness | 1 (16.7) | 1 | 2 (12.5) | 4 | 3 (13.6) | 5 |
| Suicidal ideation | 2 (33.3) | 2 | 0 | 0 | 2 (9.1) | 2 |
| RENAL AND URINARY DISORDERS | 1 (16.7) | 1 | 0 | 0 | 1 (4.5) | 1 |
| Acute kidney injury | 1 (16.7) | 1 | 0 | 0 | 1 (4.5) | 1 |
| REPRODUCTIVE SYSTEM AND BREAST DISORDERS | 0 | 0 | 1 (6.3) | 1 | 1 (4.5) | 1 |
| Ejaculation delayed | 0 | 0 | 1 (6.3) | 1 | 1 (4.5) | 1 |

AE = Adverse Event

**Table 3 – Plasma Concentrations of D-Cycloserine and Lurasidone HCl: Stage 2 Safety Population**

| Parameter | Timepoint | Statistic | lurasidone HCl | NRX-101 | All Subjects |
| --- | --- | --- | --- | --- | --- |
| D-Cycloserine (µg/mL) | Visit 2 (Day 1)/Pre-Dose | N | 6 | 15 | 21 |
|  |  | Mean (SD) | 0.000 (0.0000) | 0.000 (0.0000) | 0.000 (0.0000) |
|  |  | Median (25th, 75th) | 0.000 (0.000, 0.000) | 0.000 (0.000, 0.000) | 0.000 (0.000, 0.000) |
|  |  | Min, Max | 0.00, 0.00 | 0.00, 0.00 | 0.00, 0.00 |
|  |  | 95% CI | - | - | - |
|  |  |  |  |  |  |
| D-Cycloserine (µg/mL) | Visit 2 (Day 1)/Post-Dose | N | 6 | 14 | 20 |
|  |  | Mean (SD) | 0.000 (0.0000) | 5.709 (4.0737) | 3.997 (4.3081) |
|  |  | Median (25th, 75th) | 0.000 (0.000, 0.000) | 7.405 (0.000, 9.220) | 2.370 (0.000, 8.385) |
|  |  | Min, Max | 0.00, 0.00 | 0.00, 10.20 | 0.00, 10.20 |
|  |  | 95% CI | - | (3.357, 8.061) | (1.980, 6.013) |
|  |  |  |  |  |  |
| D-Cycloserine (µg/mL) | Visit 7 (Day 14)/Pre-Dose | N | 4 | 12 | 16 |
|  |  | Mean (SD) | 0.000 (0.0000) | 17.820 (7.8337) | 13.365 (10.4170) |
|  |  | Median (25th, 75th) | 0.000 (0.000, 0.000) | 17.950 (9.895, 23.600) | 12.300 (3.850, 20.350) |
|  |  | Min, Max | 0.00, 0.00 | 7.70, 29.80 | 0.00, 29.80 |
|  |  | 95% CI | - | (12.843, 22.797) | (7.814, 18.916) |
|  |  |  |  |  |  |
| D-Cycloserine (µg/mL) | ET/Visit 11 (Day 42)/Pre-Dose | N | 3 | 11 | 14 |
|  |  | Mean (SD) | 0.000 (0.0000) | 12.104 (8.1861) | 9.510 (8.8380) |
|  |  | Median (25th, 75th) | 0.000 (0.000, 0.000) | 9.880 (8.380, 14.800) | 9.455 (0.000, 13.700) |
|  |  | Min, Max | 0.00, 0.00 | 0.00, 28.40 | 0.00, 28.40 |
|  |  | 95% CI | - | (6.604, 17.603) | (4.407, 14.613) |
|  |  |  |  |  |  |
| D-Cycloserine (µg/mL) | ET/Visit 11 (Day 42)/Post-Dose | N | 4 | 11 | 15 |
|  |  | Mean (SD) | 0.000 (0.0000) | 24.373 (5.8653) | 17.873 (12.2080) |
|  |  | Median (25th, 75th) | 0.000 (0.000, 0.000) | 24.400 (19.200, 27.600) | 19.400 (0.000, 27.500) |
| D-Cycloserine (µg/mL) | ET/Visit 11 (Day 42)/Post-Dose | Min, Max | 0.00, 0.00 | 16.40, 36.40 | 0.00, 36.40 |
|  |  | 95% CI | - | (20.432, 28.313) | (11.113, 24.634) |
|  |  |  |  |  |  |
| Lurasidone (ng/mL) | Visit 2 (Day 1)/Pre-Dose | N | 6 | 15 | 21 |
|  |  | Mean (SD) | 0.000 (0.0000) | 0.000 (0.0000) | 0.000 (0.0000) |
|  |  | Median (25th, 75th) | 0.000 (0.000, 0.000) | 0.000 (0.000, 0.000) | 0.000 (0.000, 0.000) |
|  |  | Min, Max | 0.00, 0.00 | 0.00, 0.00 | 0.00, 0.00 |
|  |  | 95% CI | - | - | - |
|  |  |  |  |  |  |
| Lurasidone (ng/mL) | Visit 2 (Day 1)/Post-Dose | N | 6 | 14 | 20 |
|  |  | Mean (SD) | 9.813 (10.1073) | 10.366 (7.7006) | 10.200 (8.2173) |
|  |  | Median (25th, 75th) | 7.690 (0.000, 19.700) | 8.150 (4.660, 14.800) | 8.150 (4.260, 16.850) |
|  |  | Min, Max | 0.00, 23.80 | 3.10, 29.00 | 0.00, 29.00 |
|  |  | 95% CI | (-0.794, 20.420) | (5.920, 14.812) | (6.354, 14.046) |
|  |  |  |  |  |  |
| Lurasidone (ng/mL) | Visit 7 (Day 14)/Pre-Dose | N | 4 | 12 | 16 |
|  |  | Mean (SD) | 9.688 (7.1083) | 10.135 (4.8509) | 10.023 (5.2347) |
|  |  | Median (25th, 75th) | 8.070 (4.225, 15.150) | 8.960 (6.140, 11.700) | 8.960 (5.805, 11.700) |
|  |  | Min, Max | 3.51, 19.10 | 4.53, 19.60 | 3.51, 19.60 |
|  |  | 95% CI | (-1.623, 20.998) | (7.053, 13.217) | (7.234, 12.813) |
|  |  |  |  |  |  |
| Lurasidone (ng/mL) | ET/Visit 11 (Day 42)/Pre-Dose | N | 3 | 11 | 14 |
|  |  | Mean (SD) | 7.227 (1.0983) | 10.859 (5.3993) | 10.081 (5.0003) |
|  |  | Median (25th, 75th) | 7.030 (6.240, 8.410) | 10.700 (5.280, 13.500) | 8.765 (6.240, 11.600) |
|  |  | Min, Max | 6.24, 8.41 | 4.90, 22.60 | 4.90, 22.60 |
|  |  | 95% CI | (4.498, 9.955) | (7.232, 14.486) | (7.194, 12.968) |
|  |  |  |  |  |  |
| Lurasidone (ng/mL) | ET/Visit 11 (Day 42)/Post-Dose | N | 4 | 11 | 15 |
|  |  | Mean (SD) | 27.750 (12.5524) | 36.965 (22.9976) | 34.508 (20.7204) |
|  |  | Median (25th, 75th)  Min, Max | 25.350 (19.700, 35.800)  15.20, 45.10 | 31.900 (15.500, 51.700)  7.52, 75.30 | 26.500 (15.500, 49.500)  7.52, 75.30 |
|  |  | 95% CI | (7.776, 47.724) | (21.515, 52.415) | (23.033, 45.983) |

ET = Early Termination

**Appendix 3**

**Detailed Safety Results**

In the Stage 1 Safety population, all patients received the entire dose of ketamine (0.5 mg/kg, n=17) or saline (n=5). For the Stage 2 Safety population, all patients were administered an average of 58.6 oral capsules of DCS plus lurasidone or lurasidone (range 1-88; DCS plus lurasidone, n=16, mean: 59.1 capsules, range 1-88 capsules; lurasidone, n=6, mean: 57.3 capsules, range 6-82 capsules). This led to an average of 30.6 days with at least one dose of a study treatment (range 1-45 days; DCS plus lurasidone, mean: 30.9 days, range: 1-45 days; lurasidone, mean: 40.5 days, range: 3-43 days.

In the Stage 1 Safety population, 11 of the 17 ketamine-treated patients (64.7%) experienced a total of 29 AEs whereas no subject treated with saline (0%) experienced an AE. None of these 29 AEs was considered severe and no SAE was recorded. Sixteen of these 29 AEs were known to be associated with ketamine usage and were considered related to ketamine (two events each of dissociation, dizziness, euphoric mood, hypertension, and hypoesthesia, and one event each of blurred vision, diplopia, dysmetropsia, restlessness, tinnitus, and vomiting) and another 12 were considered possibly related to ketamine treatment (Table 3).

Eleven (68.8%) DCS plus lurasidone-treated patients in the Stage 2 Safety population experienced 36 total AEs, none of which were considered severe, and no SAE was experienced (Table 4). Overall, three AEs were considered related to (blurred vision, sedation, and vulvovaginal candidiasis) and 19 were considered possibly related to (four events of restlessness, three events each of akathisia and fatigue, and one event each of anorgasmia, blurred vision, ejaculation delayed, headache, hypersomnia, lethargy, sedation, somnolence, and increased weight) DCS plus lurasidone. As a result of these AEs, 12 dose reductions and three dose increases of DCS plus lurasidone were made.

Four (66.7%) lurasidone-treated patients in the Stage 2 Safety population experienced 11 total AEs (Table 4), three of these were considered severe (one event of angina pectoris and two events of suicidal ideation), and two patients experienced three SAEs (angina pectoris, suicidal ideation, and wound). However, angina pectoris was later determined to be associated with relapse of depression and the patient was transferred to inpatient psychiatric care. Overall, one AE (sedation) was considered related to lurasidone and one AE was considered possibly related to lurasidone. Nine of the 10 AEs did not result in a dose change, and one AE resulted in an unknown change.

Overall, no severe AEs, SAEs, or deaths were experienced in patients treated with ketamine 🡪 NRX-101. Three SAEs were reported in the ketamine 🡪 lurasidone group: one cardiovascular, Mean Patient Rated Inventory of Side Effects (PRISE) and Distressing PRISE did not differ between NRX-101 and lurasidone-treated groups. While ketamine predictably affected blood pressure, systolic blood pressure (SBP) and diastolic blood pressure (DBP) changes normalized within 200 and 80 minutes post-treatment, respectively. Additionally, mean pulse and weight did not change in ketamine- or DCS plus lurasidone-treated patients compared to pre-infusion baseline, while mean SBP and DBP were unchanged in DCS plus lurasidone-treated patients compared to post-infusion baseline.

The STABIL-B was not powered to prove a statistically significant difference on BARS akathisia scores. However, the study demonstrates a 1.0-point increase from baseline in the lurasidone group and a -0.2 decrease in BARS score in the NRX-101 group (d=1.1; t-test on difference; p=0.14). This trend is consistent with the preclinical data and suggests a meaningful effect in reduction of lurasidone-induced akathisia might be seen in an adequately powered trial. The anticipated sample size of the phase 2 trial has >90% power to prove the difference on BARS that was seen in STABIL-B. Therefore, the concept of a sequential treatment of a single infusion of ketamine followed by oral DCS plus lurasidone demonstrated a convincing safety profile and should be considered safe for use treating bipolar depression in patients with ASIB.
